# Histo-LCM-Hi-C Reveals 3D Chromatin Conformations of Spatially Localized Rare Cells in Tissues at High Resolution

**DOI:** 10.1093/gpbjnl/qzaf091

**Published:** 2025-09-30

**Authors:** Yixin Liu, Min Chen, Xin Liu, Zeqian Xu, Xinhui Li, Yan Guo, Daniel M Czajkowsky, Zhifeng Shao

**Affiliations:** State Key Laboratory of Systems Medicine for Cancer and Bio-ID Center, School of Biomedical Engineering, Shanghai Jiao Tong University, Shanghai 200240, China; State Key Laboratory of Systems Medicine for Cancer and Bio-ID Center, School of Biomedical Engineering, Shanghai Jiao Tong University, Shanghai 200240, China; State Key Laboratory of Systems Medicine for Cancer and Bio-ID Center, School of Biomedical Engineering, Shanghai Jiao Tong University, Shanghai 200240, China; State Key Laboratory of Systems Medicine for Cancer and Bio-ID Center, School of Biomedical Engineering, Shanghai Jiao Tong University, Shanghai 200240, China; State Key Laboratory of Systems Medicine for Cancer and Bio-ID Center, School of Biomedical Engineering, Shanghai Jiao Tong University, Shanghai 200240, China; State Key Laboratory of Systems Medicine for Cancer and Bio-ID Center, School of Biomedical Engineering, Shanghai Jiao Tong University, Shanghai 200240, China; State Key Laboratory of Systems Medicine for Cancer and Bio-ID Center, School of Biomedical Engineering, Shanghai Jiao Tong University, Shanghai 200240, China; State Key Laboratory of Systems Medicine for Cancer and Bio-ID Center, School of Biomedical Engineering, Shanghai Jiao Tong University, Shanghai 200240, China

**Keywords:** Spatial-omics, Hi-C, Tissue, Rare cell, Laser capture microdissection

## Abstract

Understanding chromatin structure is essential to delineate the mechanisms underlying genomic processes. However, while methods to obtain such information from cells *in vitro* are widely available, there is presently a significant lack of techniques that can acquire these data from cells in tissue. Such a capability is critical to determine the dependence of the local tissue environment on cell function. Further, this ability is particularly necessary for cells that constitute a significant minority of the total tissue population, as they are often obscured in data dominated by more abundant cell types. Here, we developed a high-throughput chromosome conformation capture (Hi-C)-based method, Histological laser capture microdissection Hi-C (Histo-LCM-Hi-C), to enable the characterization of chromatin architecture of phenotype-defined, spatially localized cells within intact tissue sections from as few as ∼ 300 cells. We demonstrated the effectiveness of this approach by generating the first 3D Hi-C map of liver-resident macrophages, the Kupffer cells (KCs), a minor cell population in the normal liver. As expected, owing to their relative rarity, these KC maps were significantly different from those obtained from the whole liver, revealing distant contacts between putative enhancers and genes involved in key KC functions, as well as significant differences from those of *in vitro* induced bone marrow-derived macrophages. We anticipate that this method will prove to be an indispensable technique in the growing repertoire of methodologies for characterizing the genomic properties of cells within their native environment.

## Graphical abstract

**Figure:**
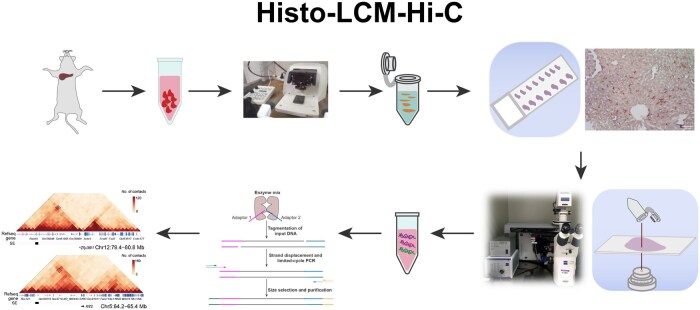

## Introduction

It has long been recognized that organs are composed of cells with a wide range of phenotypes, whose functions are tightly coordinated to ensure proper organ function. A critical aspect of this coordination is the precise spatial organization of cells within the tissue: the interactions between cells and their local milieu profoundly influence gene expression and cellular function [1–3]. The recent proliferation of spatial-omics technologies has revealed a tremendous amount of information, significantly advancing our understanding of cellular function in native tissues [4–6]. However, among these spatial-omics methods, there is a notable lack of techniques that provide genome-wide views of chromatin architecture, even though 3D genome conformation is now established to play a fundamental role in many genomic processes, most notably transcription [7,8]. Understanding of chromosome structure is now recognized as ultimately essential for identifying contacts between enhancers and promoters, which play a pivotal role in gene expression regulation [9].

To date, high-throughput chromosome conformation capture (Hi-C) is one of the most common methods for studying chromatin architecture *in vitro* [10]. With this method, structures over a range of genomic scales have been identified, from point-to-point contacts that define loops [11] to more extended regions of enhanced contacts that span hundreds to thousands of base pairs called topologically-associated domains (TADs) [12], and to much longer-range contacts of broad regions that define compartments [13]. The Hi-C method employs a series of enzymatic steps that are highly effective in cultured cells but far less effective when applied to intact tissue blocks, owing to reduced enzyme diffusion within the dense tissue matrix. Furthermore, the Hi-C maps obtained from such samples would be averages over the entire cell population in the tissue, regardless of their positioning or phenotype. To our knowledge, there is presently no established method to obtain Hi-C maps from spatially defined cells within intact native tissue.

With this in mind, we reasoned that laser capture microdissection (LCM) is capable of isolating specific cells directly from tissue sections [14,15]. When combined with an appropriate marker, LCM allows for the ready identification and selection of even rare cells, thereby enabling direct and reliable characterization. Notably, many spatial-omics methods developed to date struggle to characterize rare cells within tissues, a well-recognized challenge in the field [4]. Here, we present a new protocol, Histological LCM Hi-C (Histo-LCM-Hi-C), that uses cells specifically identified by standard histological staining techniques to acquire high-quality Hi-C data from as few as 300 cells. We demonstrated the power of this approach by generating the first Hi-C maps of Kupffer cells (KCs) directly isolated from mouse liver sections. These maps reveal previously unrecognized enhancer–promoter contacts specific to KCs and are associated with genes that play pivotal roles in KC function. We anticipate that this methodology will find broad application within the growing arsenal of spatial-omics methods used to study the functions of native tissues.

## Method

### Preparation of the mouse tissue sample

The samples were obtained from age- and sex-matched 12- to 13-week-old male C57BL/6 mice (Jie Si Jie Laboratory Animals, Shanghai, China), including four mice for liver samples and two for brain samples. Mice were housed under *ad libitum* feeding conditions and maintained on a reversed light–dark cycle. Mice were euthanized by cervical dislocation 6 h after lights-off. The liver was then freshly dissected, washed with ice-cold phosphate-buffered saline (PBS), and cut into small blocks. For the optimized method, the tissue blocks were fixed in 1% paraformaldehyde at 17°C for 4 h, followed by quenching with 0.125 M glycine for 15 min at room temperature. The blocks were then stored at 4°C.

### *In situ* Hi-C in tissue sections

The tissue blocks were placed in ice-cold PBS and cut into 8 to 10 µm thick sections using a vibratome (speed: 0.50 mm/s, amplitude: 0.75 mm). Hi-C was then performed following a published method [16] with modifications. For the optimized method, the sections were incubated in lysis buffer [10 mM Tris-HCl, pH 8.0; 10 mM NaCl; 0.2% Tween-20; 1% Protease Inhibitor Cocktail (Catalog No. P8340, Sigma-Aldrich, St. Louis, MO)] on ice for 20 min, followed by washing with NEBuffer 2 (Catalog No. B7002S, NEB, Ipswich, MA). The sections were then treated with sodium dodecyl sulfate (SDS) (final concentration: 1.2%) at 65°C for 1 h, followed by incubation with Triton X-100 (final concentration: 1%) at 37°C for 15 min. The samples were digested overnight at 37°C with MboI restriction enzyme (Catalog No. R0147, 5 U/ml, NEB), end-repaired with Klenow enzyme (Catalog No. M0210, NEB) at 37°C for 2 h, and ligated using T4 ligase (Catalog No. M0202, NEB) at 25°C for 8 h. Finally, the samples were treated with Exonuclease III (Catalog No. M0206, 100 U/ml, NEB) for 5 min at 37°C and stored at −80°C until further processing. Analysis of the gel data was performed using ImageJ (v1.54p).

### Labeling of KCs

The sections were washed three times with ddH_2_O for 5 min and then incubated with 3% hydrogen peroxide at room temperature for 10 min. The sections were blocked with 5% BSA and washed with PBS. Next, the sections were incubated with the primary antibody, anti-rabbit F4/80 antibody (Catalog No. 70076s, Cell Signaling Technology, Danvers, MA), at 4°C overnight. Afterward, the sections were washed with PBST (1× PBS with 0.1% Tween). The sections were then incubated with goat anti-rabbit IgG H&L (HRP)-conjugated antibody [Immunohistochemistry Kit (Catalog No. G1215-200T, Servicebio, Wuhan, China)] for 2 h at room temperature. The sections were washed three times with PBS for 5 min each. Then, 50 to 100 µl of DAB working solution was added, and the mixture was incubated at room temperature for several minutes until the desired color developed under the microscope. Following this, the sections were washed with ddH_2_O to terminate color development. Finally, the sections were counterstained with hematoxylin for about 1 to 2 min and washed with ddH_2_O for 2 min. If the tissue sections were used for LCM selection, the counterstaining step was omitted.

### LCM

Following histo-labeling, the sections were placed on a polyethylene terephthalate (PET)-membrane-covered slide (Catalog No. 415190-9051-000, Carl Zeiss, Jena, Germany), and the KCs were micro-dissected at the single nuclear region level using the Zeiss PALM Micro Beam LCM system (Zeiss Microimaging, Munich, Germany) housed in a Plexiglas housing. Briefly, the perimeter of the chosen cell was first finely cut with a focused laser beam with a resolution of less than 1 µm. This resolution ensures that only the chosen cell, and in particular its nucleus, is collected, not that of the adjacent cells. After this cutting, the isolated region was ejected into the adhesive cap of the collection tubes (Catalog No. 415190-9191-000, Carl Zeiss) using a pulse from a UV laser. We continued this process for the KCs, collecting material one cell at a time until sufficient materials were accumulated. To avoid external contamination, the LCM system was housed in a dedicated plexiglass chamber, which isolates all sorting, dissection, and ejection steps from the external environment. We also inspected the adhesive caps of the collection tubes under a phase-contrast microscope to confirm the absence of debris or foreign particulates, and subsequently placed the tube in a Biological Safety Cabinet (1300 Series A2, Thermo Fisher Scientific, Marietta, OH) to perform the subsequent experimental steps.

### DNA extraction and Hi-C library preparation

The collected sample was incubated with proteinase K (1 mg/ml) and SDS (1%) at 65°C for 3 h, followed by the addition of sodium chloride to a final concentration of 500 μM, and incubated at 65°C overnight. All steps from the opening of the adhesive cap to buffer addition were performed on a dedicated workbench to prevent environmental contamination. DNA was captured using 1.8× Agencourt AMPure XP beads (Catalog No. A63881, Beckman, Brea, CA). The DNA was then resuspended in 10 mM Tris-HCl, and the Tn5 enzyme [TruePrep DNA Library Prep Kit V2 for Illumina (Catalog No. TD502/TD503, Vazyme, Nanjing, China)] was added to fragment the DNA and attach adaptors. Biotin-labeled chimeric molecules were captured by incubating with Dynabeads^®^ MyOne™ Streptavidin C1 beads (Catalog No. 65001, Thermo Fisher Scientific, Vilnius, Lithuania) at room temperature for 2 h. After incubation, the beads were washed with 1× Tween wash buffer (0.05% Tween 20, 1 M NaCl, 0.5 mM EDTA-NaOH, 5 mM Tris-HCl, pH 8) on a Thermomixer at 55°C for 5 min, repeated four times. After washing, the DNA on the beads was amplified using the TruePrep™ DNA Library Prep Kit V2 (Catalog No. TD503, Vazyme). Finally, 0.55× and 0.75× Agencourt AMPure XP beads were used to select DNA fragments of the appropriate size for sequencing. All libraries in this study were sequenced on the NovaSeq 6000 platform. Standardized protocols were implemented for tissue fixation, LCM, Hi-C library preparation, and downstream data analysis for all samples.

### Hi-C data analysis

Hi-C reads were iteratively mapped to the mm10 genome using Bowtie2 (v2.1.0) with a step size of 25 bp. Reads that were mapped to multiple genomic locations or had low mapping quality (MAPQ; < 30) were removed. PCR duplicates were further removed. Read pairs identified as originating from the same fragment (*i.e.*, pairs mapped to the same restriction fragment), nonspecific ligations (fragments with sizes above 600 bp), dangling ends (inward-facing read pairs less than 1 kb apart), and self-ligations (outward-facing read pairs less than 25 kb apart) were excluded. Valid pairwise reads were retained for each cell. Juicer tools (v1.19.02) were used to create the .hic file and perform KR normalization. The similarity between Hi-C datasets was calculated using HiCRep [17]. Compartment analysis was performed using Juicer tools at a 100-kb bin size (except where noted), with the eigenvector corrected based on gene density. Regions with values greater than 0 were considered A compartments, and those with values less than 0 were considered B compartments. TADs were identified using TopDom [18] at a 40-kb bin size and a window size of 5; only regions with *P* < 0.05 were retained. For comparisons between samples, TAD borders within one 40-kb bin of each other were considered co-localized. The valid pairwise reads per cell were calculated by dividing the total valid pairwise reads of one library by the number of cells in the library, the latter of which was determined based on the quantity of DNA. FitHiC2 [19] was used to identify significant Hi-C contacts. All bins with very low contact frequencies (namely, bins that contact fewer than 3% of the other bins) were excluded from this analysis. This software builds its empirical null model based on the input data and does not require a strict sequencing depth or resolution [19]. For all analyses, based on our sequencing depth, we used a bin size of 20 kb to ensure the greatest confidence in the obtained results. For calculations of contacts with super-enhancers (SEs), we used a *q*-value threshold of 0.005 to select the most significant contacts with these functional elements. We used Mustache [20] to identify loops in the Hi-C data, setting the sparsityThreshold to 0.88 and a *P* value threshold of 0.05. We also used diffHic [21] to identify local differences in the Hi-C contact maps at a 40-kb bin size, with |log_2_ FC| greater than 0.4 and a *P* value of 0.05 as thresholds. The published Alpha Mouse Liver 12 (AML12) cell Hi-C data, brain Hi-C data, and bone marrow-derived macrophage (BMDM) Hi-C data are available in Gene Expression Omnibus (GEO) under the accession numbers GSE136306, GSE34587, and GSE115524, respectively. The published liver Hi-C data are available at GEO with accession numbers GSE65126, GSE104129, and GSE58752.

### Identification of SEs

SEs were identified using the Rank Ordering of Super-Enhancers (ROSE) algorithm [22,23]. This approach was employed to delineate transcriptional regulatory elements throughout the genome, specifically focusing on regions located at least 3 kb away from transcription start sites (TSSs). To achieve this, adjacent H3K27ac ChIP-seq signals were integrated using the parameter “-t 3000.” Subsequently, the highest-ranked regions were classified as SEs in the KCs. The published H3K27ac ChIP-seq data for KCs are available at GEO with the accession number GSE216164.

### RNA-seq data analysis

Raw reads were processed with Trimmomatic (v0.35) to remove low-quality reads using the following parameters: “SLIDINGWINDOW:3:20 LEADING:20 TRAILING:20 MINLEN:30”. rRNA reads were removed using SortMeRNA. FastQC (v0.11.5) was used to assess the quality of the clean reads. The clean reads were then mapped to the mm10 genome using HISAT2 (v2.0.5). Gene expression levels were calculated as transcripts per million (TPM) using StringTie (v1.3.3), with only mRNAs included. The Mus_musculus.GRCm38.96.chr_mRNA.gtf file was used and downloaded from http://ensemblgenomes.org/. TSS information was obtained from the same .gtf file. Gene Ontology (GO) analysis was performed using ShinyGo [24] and Metascape [25]. The published KC RNA-seq data and liver (hepatocyte) RNA-seq data are available at GEO with the accession numbers GSE186327 and GSE150835, respectively.

## Results

### Overview of Histo-LCM Hi-C

**Figure 1:**
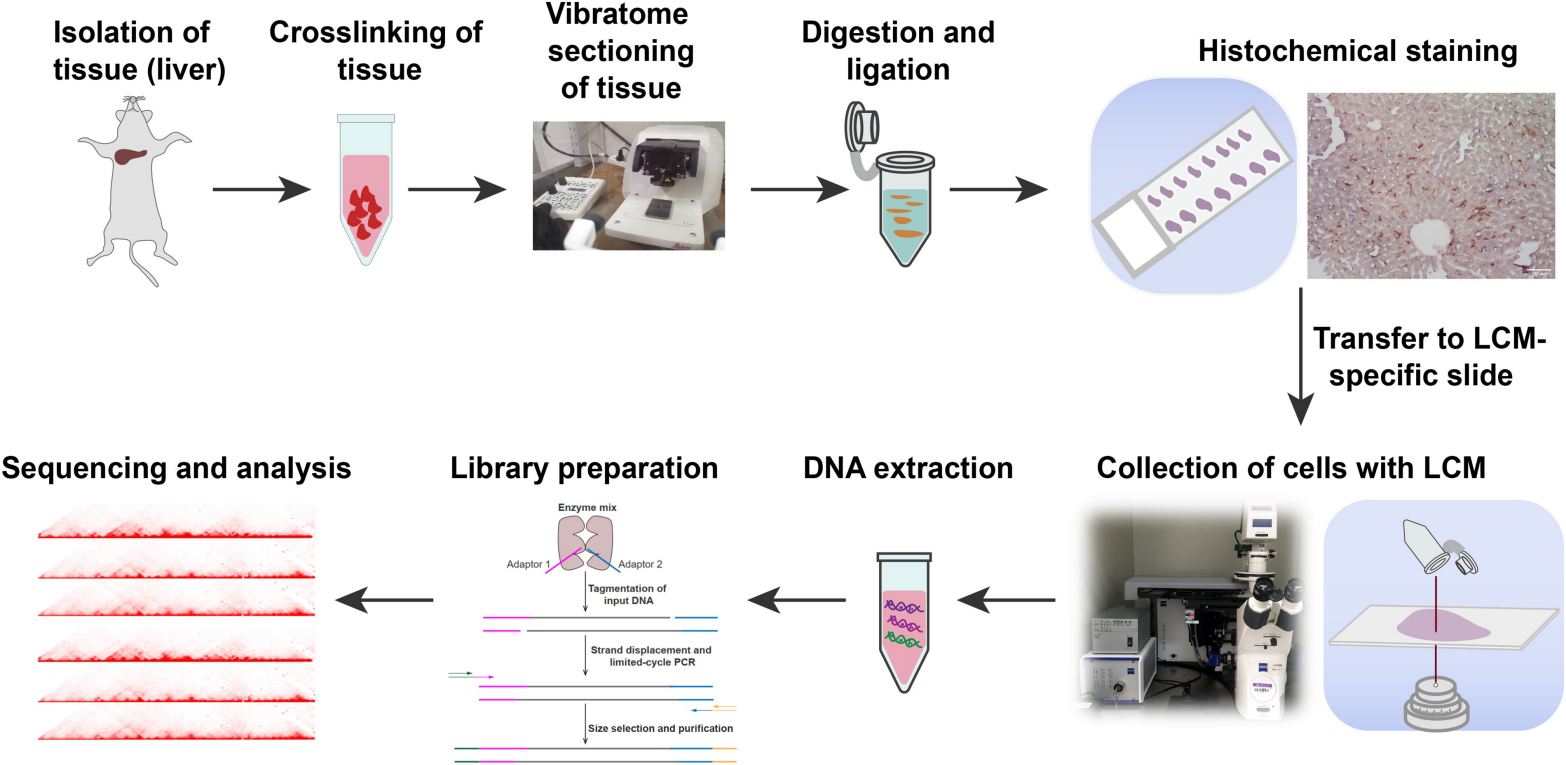
Overview of the Histo-LCM-Hi-C method Histo, histological; LCM, laser capture microdissection; Hi-C, high-throughput chromosome conformation capture.

The workflow of this approach is outlined schematically in **Figure 1**. Briefly, dissected tissues are first crosslinked with paraformaldehyde and then sectioned with a vibratome. The sections are then suspended in a solution, and the digestion and ligation steps of *in situ* Hi-C are performed. After completing these steps, the treated sections are deposited onto glass slides, histochemically stained, transferred to the specific slides used for LCM, and then LCM is used to isolate specific cells from the stained sections. Finally, the Hi-C library is prepared from the isolated materials using methods slightly altered from published protocols [26], and the DNA is sequenced.

### Optimization of the Hi-C protocol for tissue sections

As described in the Introduction section, performing the enzymatic reactions of Hi-C with an intact whole tissue is generally challenging owing to the reduced diffusion of enzymes through the densely cross-linked tissue. Consequently, most prior Hi-C experiments involving tissues have dissociated the constituent cells into single cells or nuclei suspensions and performed the Hi-C procedure with these suspensions. However, such approaches prohibit any opportunity to understand the spatial dependence of cell properties within the tissue. Moreover, simply isolating cells from the tissue can alter chromatin structure [27]. Therefore, we sought to develop a Hi-C-based method to directly characterize defined cell types within the tissue.

To this end, we first focused on identifying effective conditions for crosslinking the sample, as this step is critical not only for ensuring a native chromatin structure but also for allowing efficient restriction/ligation during the Hi-C protocol in the tissue section. To identify these conditions, we used micrococcal nuclease (MNase) as a general measure of chromatin accessibility to enzymatic digestion [28]. In particular, we used the extent of digestion to mono-nucleosome-spaced bands as an indicator of a practically useful level of crosslinking. We noted that it is essential to achieve uniform and sufficient crosslinking across the sample, which is generally more challenging in a tissue/organ than in cultured or isolated cells. With blocks of mouse liver tissue, we found that typical tissue crosslinking conditions (4% paraformaldehyde for several days at 4°C [29]) resulted in samples that were too heavily crosslinked for MNase digestion. In contrast, milder crosslinking conditions (1% paraformaldehyde for 10 min at room temperature, as used in standard Hi-C) produced smeared bands after MNase digestion (Figure S1A), indicating insufficient crosslinking. Thus, we examined the effectiveness of fixation for different times (2 h, 3 h, or 4 h) and at different temperatures (4°C, 17°C, and room temperature), monitoring the level of MNase digestion and tissue mechanical strength. Ultimately, we found that short fixation times (< 3 h) were not effective, as they resulted in overly soft tissues that could not be efficiently sectioned. Moreover, low temperatures failed to preserve chromatin integrity. Hence, we settled on 1% paraformaldehyde for 4 h at 17°C, which yielded clear nucleosome-sized MNase digestion bands, thus indicating well-crosslinked samples (Figure S1B; see the Method section). We also compared the results obtained using this fixation protocol with cultured AML12 cells in relation to those obtained with the conventional fixation protocol and found excellent agreement (Figure S1C).

Under these conditions, we next focused on identifying the optimal section thickness for the Hi-C reactions. Since the size of a typical nucleus is in the range of 8 to 15 μm, we first examined sections with thicknesses between 10 μm and 50 μm to determine the extent to which the restriction enzyme used in the Hi-C protocol (MboI) can digest the genomic chromatin. We performed this digestion and all Hi-C steps with freely floating sections so that the enzymes could access them equally from all sides. However, we were surprised to find that the restriction digestion reaction was poor for thicknesses greater than 10 µm, even though the aforementioned MNase digestion assay was effective at these thicknesses (Figure S2A–C). We speculate that this reduced effectiveness may reflect lower MboI diffusion relative to MNase, owing to its larger molecular size [30,31]. Nonetheless, for this method, we used section thicknesses of 10 µm.

Next, we optimized the key parameters of the Hi-C procedure. We found that increasing the SDS treatment time improved digestion efficiency (Figure S2D; also see the Method section). In addition, extending the times for end-repair and T4 ligase treatment significantly increased the ligation efficiency, yielding a sharp high-molecular-weight band in the agarose gel as expected (compare results in Figure S3A and B). The library produced under these conditions was also of high quality (Figure S4A). Finally, we confirmed that these experimental conditions were also effective with a different tissue, namely, the mouse brain cortex (Figure S3C).

**Figure 2:**
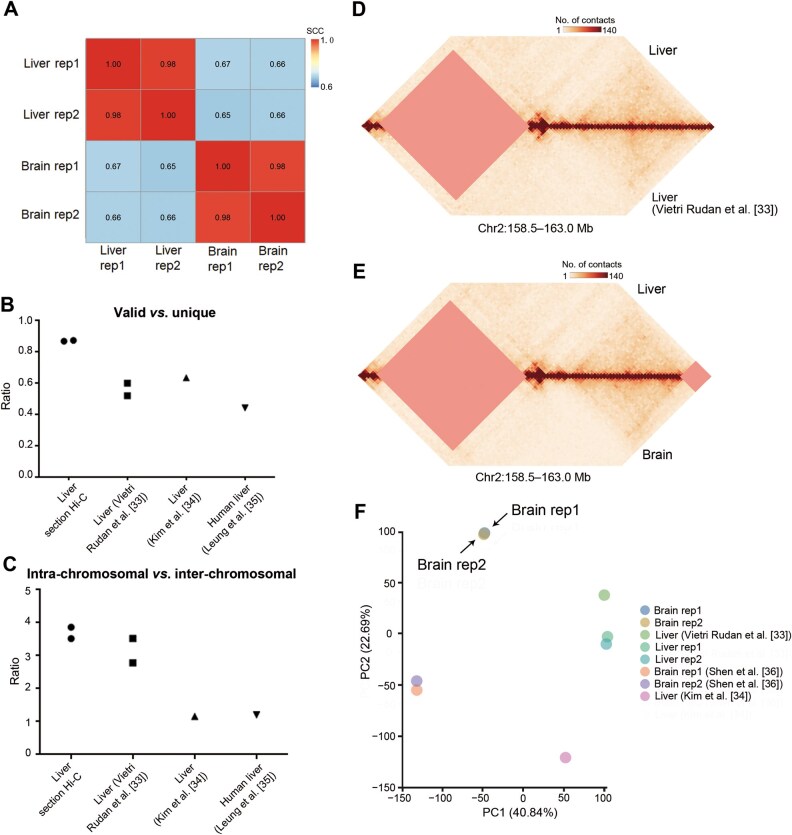
Comparison of the Hi-C data from brain and liver tissue sections with each other and existing datasets **A**. Correlation results between the tissue section Hi-C data of liver and brain replicates. **B**. The proportion of valid pairwise reads in the liver tissue section Hi-C compared with the published mouse liver cell standard Hi-C data. **C**. The ratio of intra-chromosomal pairwise reads to inter-chromosomal pairwise reads in liver tissue section Hi-C, as well as that for the published mouse liver cell standard Hi-C data. **D**. and **E**. Visualization of contact maps generated from tissue section Hi-C of liver and published mouse liver cell standard Hi-C data (D), as well as tissue section Hi-C of brain (E). Resolution: 40 kb. **F**. Comparison of the similarity of the data obtained from the indicated samples based on their corrected PC1 values (where PC1 values are typically used in compartment analysis) [33,34,36]. SCC, stratum-adjusted correlation coefficient; PC, principal component; rep, replicate; Chr, chromosome.

With these technical issues optimized, we examined the overall effectiveness of our Hi-C protocol with mouse liver and brain sections. As shown in Figure S4A and B and **Table 1**, the sequenced libraries prepared with our protocol were of high quality and comparable to those of cultured AML12 mouse hepatocyte cells obtained with the standard Hi-C protocol [16]. In addition, the maps obtained with our method were highly reproducible, exhibiting a stratum-adjusted correlation coefficient (SCC, bin size 500 kb) [17] of 0.98 between biological replicates (**Figure 2**A). By contrast, the SCC between the liver and brain sections was notably lower, at 0.64 (Figure 2A). We noted that the SCC between the liver and AML12 cells was also low (SCC of 0.55), suggesting that this cell line may not, in fact, accurately reflect the native hepatocytes in the tissue (which comprise approximately 65% to 70% of the cells in the liver [32]).

**Table 1:** Hi-C data from tissue sections and the AML12 cell line.

Parameter	Brain rep1	Brain rep2	Liver rep1	Liver rep2	AML12 rep1	AML12 rep2
No. of raw reads	160,848,154	175,003,865	53,552,169	194,377,862	213,721,058	195,013,809
No. of reads with MAPQ ≥ 30	99,040,570	107,767,081	38,695,055	140,320,344	141,943,465	126,168,943
No. of reads after PCR duplicate removal	76,168,576	92,854,661	27,893,906	100,608,487	101,532,358	108,059,298
No. of reads filtered based on restriction enzyme sites	25,537,401	29,653,961	3,640,711	12,588,686	38,130,058	31,621,835
No. of valid reads	50,631,175	63,200,700	24,253,195	88,019,801	63,402,300	76,437,463
No. of *cis*-interactions	40,822,535	52,653,103	18,883,608	69,929,164	42,289,461	52,565,598
No. of *trans*-interactions	9,808,640	10,547,597	5,369,587	18,090,637	21,112,839	23,871,865

*Note*: Hi-C, high-throughput chromosome conformation capture; MAPQ, mapping quality; AML12: Alpha Mouse Liver 12.

We also found that the quality of our liver Hi-C data was comparable to or better than that of previously published data from mouse liver cells [33–35] (Figure 2B and C) and AML12 cells, the latter two of which were obtained using standard Hi-C (Figure S5). In addition, over large genomic distances (namely, at a bin size of 500 kb), our liver section data were highly correlated with previously published data (SCC of 0.95 and 0.92). However, we found that, over short distances (bin size of 40-kb), this correlation was lower (SCC of 0.90 and 0.77), suggesting differences in structure at the TAD/sub-TAD scale (Figure S6A and B). Such differences between our liver section data and previously published work were also apparent by direct inspection of the Hi-C contact maps (Figure 2D**)**. We noted that such differences were less frequent than those between the liver and brain samples (Figure 2E). Examination of these maps at the compartment level (obtained at a bin size of 100 kb) also showed a similar trend, with clear differences between the liver section and the published liver work (Figure 2F). A similar finding was also observed for the brain tissue section Hi-C data and published brain Hi-C data [36] (SCC of 0.89 and 0.81 for bin sizes of 500 kb and 40-kb, respectively) (Figure 2F, Figure S6A and B). We noted that these published bulk cell Hi-C data were obtained from tissues that were first dissociated into cell or nuclear suspensions before performing the Hi-C steps, in contrast to our method with tissue sections. Hence, the cell/nuclei isolation step may also have contributed to the observed differences. Overall, these results demonstrate that our tissue section Hi-C protocol is highly effective and may provide more faithful depictions of chromatin conformations in tissues.

### Histo-LCM-Hi-C for the characterization of defined cells in tissues

Having established our tissue section Hi-C procedure, we next sought to isolate and characterize specifically defined cells using histological staining and LCM. To demonstrate the power of this method, we focused on characterizing the resident liver macrophages, the KCs, which comprise a significant minority of the total liver cells in mice (8% to 9%) [37]. Therefore, the chromatin conformation of these cells is not expected to be evident in the data from bulk liver tissue samples. Indeed, we noted that, to date, there is no data on chromosome conformation of these cells in the literature.

**Figure 3:**
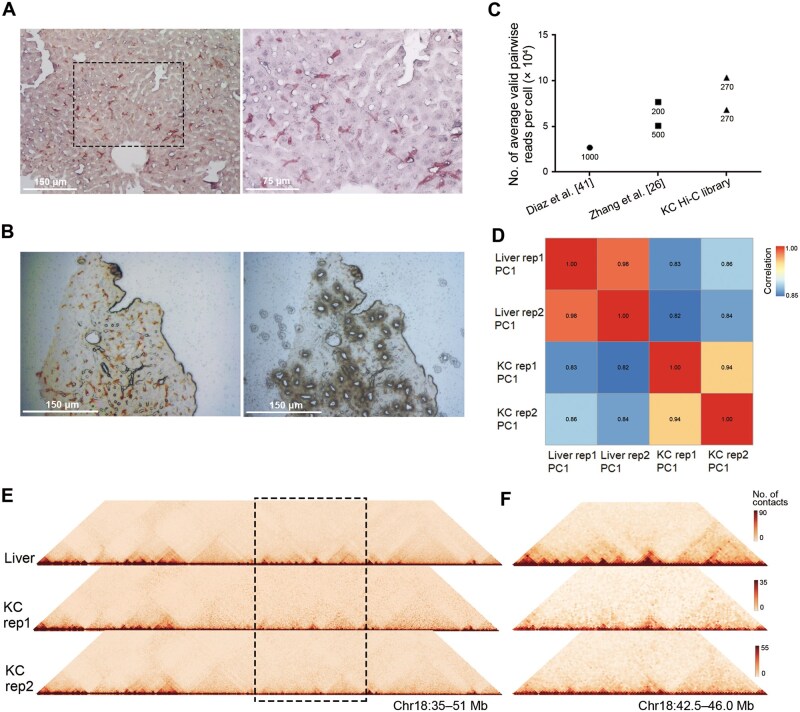
Generation of high-quality Hi-C maps for specific rare cells in the liver **A**. Immunohistochemistry images of KCs in the mouse liver section, using anti-F4/80 to label the KCs and Hematoxylin to label the nucleus. The right panel is a magnified view of the dotted-boxed region in the left image. Scale bar: 150 μm (left) and 75 μm (right). **B**. A typical stained section before (left) and after (right) laser microdissection. Scale bar: 150 μm. **C**. Average valid pairwise reads per cell of the KC Hi-C libraries compared with previously obtained low-input Hi-C data [26,41]. The numbers in the figure indicate the number of cells in each Hi-C library. **D**. Correlation results of the PC1 values from replicates derived from tissue section Hi-C libraries of the liver and KCs. **E**. Comparison of the contact maps generated from tissue section Hi-C of liver and KCs. **F**. A magnified region of the heatmap within the dotted boxed region of “E”. Resolution: 40 kb. KC, Kupffer cell.

We first verified that the staining and LCM isolation did not significantly affect the Hi-C results compared with our tissue section results (SCC of 0.95) and yielded mapping data of comparable quality (Table S1). We then used LCM to select individual KCs (**Figure 3**A and B), using the F4/80 protein as a marker [38]. To be clear, owing to the randomness of the sectioning with respect to the positions of the cells and the similarity in size between a typical nucleus and the section thickness, we expected that each nuclear region identified in our sections was not an intact nucleus but only a portion of the nucleus. Indeed, we found that ∼ 600 of these nuclear regions yielded only about 1.63 ng of DNA, which corresponds to about 270 intact cells. Experimentally, we found that we required ∼ 600 of these regions for the construction of an effective library for next-generation sequencing. In particular, examination of the DNA fragment size distribution revealed no or an abnormal distribution from the libraries obtained fewer than 600 nuclear regions (Figure S7A and B). Furthermore, sequencing at a relatively low depth to examine material quality (namely, total reads < 15 million) showed an extremely high proportion of duplicate reads (> 80%), revealing a very low library complexity. A simple calculation suggested that this lower cell limit reflects a limitation of the Hi-C method, rather than, for example, significantly inefficient enzymatic reactions under our conditions. Specifically, based on the total valid pairwise reads of one library obtained in our experiment (and assuming there were indeed effectively 270 cells), we found that our coverage per cell (103 thousand valid pairwise reads) was comparable to that observed from typical single-cell Hi-C studies (where there are clearly no access issues to the added enzymes) [39,40] (Table S2). Thus, our method exhibits a comparable level of efficiency to at least most single-cell Hi-C assays.

We obtained two libraries from a single mouse and found that the Hi-C maps from each sample were highly correlated (SCC of 0.96) (Figure S8A). To further validate the reproducibility of these results, we obtained four additional libraries from a different mouse. We found that these were also highly correlated with each other and with those of the first mouse (SCC of ∼ 0.93) (Figure S8A). Notably, the KC libraries obtained from as few as ∼ 300 cells exhibited higher valid interaction ratios than those from standard Hi-C protocols, underscoring the enhanced sensitivity of our method (Figure S8B). We found that at comparable sequencing depths, the data obtained using Histo-LCM-Hi-C were on par with other published low-input Hi-C methods, further demonstrating the effectiveness of our approach [26,41] (Figure 3C; Table S3).

Data from each mouse were pooled together to obtain a KC replicate map (for each mouse). Examination of these maps at the level of compartments showed that the KC samples were much more similar to each other than to the total liver sample (Figure 3D). This was also evident by direct inspection of the Hi-C maps (Figure 3E and F). Owing to the high consistency between the two biological KC replicates, we combined the replicate data to improve the resolution of the final map for more detailed analyses below, ultimately obtaining a map at 40-kb resolution.

Notably, our method achieved this resolution with only ∼ 3000 nuclear regions (∼ 1500 intact cells), whereas standard Hi-C typically uses 10^6^ cells for comparable resolution. To provide insight into the relationship between cell number and resolvable structural features, we further examined the maps obtained from 300-cell, 750-cell, and 1500-cell samples. The resolutions of these samples, following the conventional definition of resolution (80% of bins containing 1000 reads), were 200 kb, 80 kb, and 40-kb, respectively. We calculated the number of A/B compartments at a bin size of 200 kb for the 300-cell, 750-cell, and 1500-cell samples and found similar numbers of compartments for each (Figure S9A). For the 750-cell and 1500-cell samples, we also calculated the number of A/B compartments at a bin size of 100 kb (the typical bin size used for compartment identification) and found a slightly higher number of compartments (Figure S9A), as expected with a smaller bin size. For the TADs, we compared the numbers from the 750-cell and 1500-cell samples at a bin size of 80 kb and found ∼ 3500 TADs in both cell samples (Figure S9B). We also calculated the TAD number at a 40-kb bin size with the 1500-cell sample and found ∼ 5300 TADs. Thus, the majority of TADs can be resolved even with only the 750-cell sample. Finally, for loops, we examined the 750-cell and 1500-cell samples at an 80-kb bin size and the 1500-cell sample at a 40-kb bin size using Mustache [40]. We found that only about half of the loops observed in the 1500-cell sample at a 40-kb bin size were observed in the 750-cell sample at an 80-kb bin size, clearly reflecting the improved signal-to-noise ratio and precision in the larger cell-number sample (Figure S9C). Overall, these results suggest that with 300 cells, we can obtain reliable information about compartments, while 750 cells would be needed for reliable measurements of both compartments and TADs, and 1500 cells would provide the most structural information about compartments, TADs, and loops.

We also examined our 1500-cell KC map to evaluate the extent to which it resolved true chromatin interactions from background noise, compared to a larger cell-number sample (*i.e.*, the liver). Using FitHiC2, we found that the data from both liver and KC samples exhibited a similar level of true contacts (Table S4) and both exceeded those in other published work (Figure S10). We also examined our results after down-sampling the reads of small-cell-number KC datasets, but found no significant difference from the results without down-sampling (Table S4).

### Analysis of the KC chromatin architecture reveals mechanistic details underlying its functioning

KCs are the resident macrophages of the liver, and as such, play crucial roles in immune reactions and liver-specific metabolic functions [42]. Their primary functions include clearing microorganisms and cellular debris from the blood, removing aging red blood cells, providing a barrier to prevent various pathogens and their toxic byproducts (such as endotoxins) from entering the systemic circulation, and supporting protein and lipid metabolism [43].

Overall, our final KC Hi-C map exhibited many features similar to those observed in previous *in vitro* Hi-C studies of other cell types [44,45], such as, for example, showing ∼ 5000 TADs with a median size of 400 kb (Table S5). We were particularly interested in examining regions of the maps that differ between KCs and the whole liver, as these might reveal novel details of gene regulation within KCs. Owing to the established relationship between compartment type and gene expression, we first compared these maps at the compartment level (determined at a bin size of 100 kb), finding that 5% (1270) of regions were A in KCs and B in the whole liver, and 6% (1671) were B in the KCs and A in the liver (Figure S11A). For regions unchanged in compartment type, we found differences in the overall number of contacts within each compartment, which indicates significantly different levels of compaction between these samples (Figure S11B).

**Figure 4:**
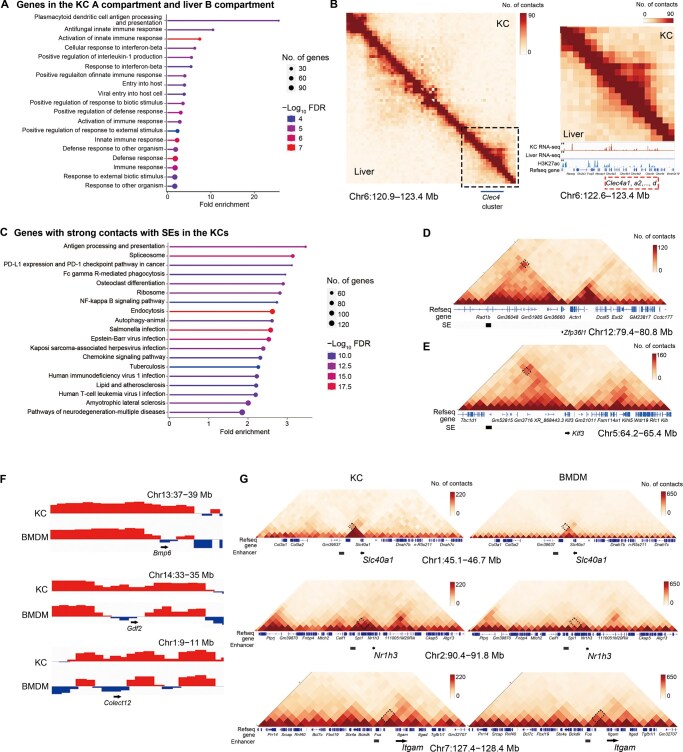
KC-specific chromatin structures and comparison with BMDM **A**. GO analysis results of the genes in the KC A compartment and liver B compartment. **B**. Visualization of contact maps generated from tissue section Hi-C of the KCs (top) and liver samples (bottom) in Chr6, from 120.9 Mb to 123.4 Mb, resolution: 40 kb. The right panel shows a magnified heatmap of the boxed region in the left panel from 122.6 Mb to 123.4 Mb, together with RNA-seq of KCs and liver tissue, H3K27ac ChIP-seq data of the KCs, and Refseq genes. **C**. GO analysis results of the genes with strong contacts (from FitHiC2, *q*-value < 0.005) with SEs in the KCs. **D**. and **E**. Visualization of contact maps showing the SE-gene contacts with *Zfp36l1* (D) and *Klf3* (E), resolution: 40 kb. In both images, the black bar indicates the position of the SE, together with Refseq and SE. **F**. Plots of the PC1 values reflecting compartment-level interactions of KCs (upper panels) and BMDMs (lower panels). Positive values (red) reflect A compartments and negative values (blue) reflect B compartments. **G**. Visualization of the contact maps of three exemplary regions, showing stronger contacts between putative enhancers [53] (the black bar) and genes with prominent functions in either KCs or BMDMs, together with the Refseq genes. Resolution: 40kb. BMDM, bone marrow-derived macrophage; RNA-seq, RNA sequencing; SE, super-enhancer.

We found that the regions designated as A compartments in the KCs and B compartments in the liver were enriched for immune-related genes. In particular, we observed enrichment for genes involved in pathways such as the activation of the innate immune response and positive regulation of lymphocyte proliferation (**Figure 4**A; Table S6). For example, we found that both *Ccl2* and *Il16* were located in the A compartments in the KCs and B compartments of the liver; both of these proteins are pro-inflammatory cytokines that are chemotactic for monocytes, CD4^+^ T lymphocytes, and eosinophils [46,47]. Thus, these results suggest that the expression of genes of this important pathway in KCs for hepatic immunity is indeed regulated by specific chromatin conformations. Likewise, regions that are B compartments in the KCs and A compartments in the liver were more often associated with genes involved in core metabolic functions of the liver, which are not expressed in the KCs, likely related to their location within the B compartments in the KCs (Figure S11C).

We performed diffHic [21] to identify regions of local structural differences between KC and total liver samples. Overall, we found that ∼ 84% of the genome (at 40-kb bins) exhibited at least one change in contact with another region of the genome (Table S7). One of the most significantly different loci overlapped the *Clec4* gene cluster, whose members are specifically expressed in KCs [48,49] and are involved in phagocytosis and production of inflammatory cytokines and chemokines in KCs [50–52]. We found that this locus formed a separate TAD in the KCs that was completely absent in the total liver sample (Figure 4B), which may ensure effective contacts between enhancers and the *Clec4* promoters in the KCs. Another top diffHiC locus overlapped the *Ifi200* family gene cluster, which exhibits significantly greater contacts with a putative enhancer [53] in the KCs than in the liver (Figure S12A and B). This gene cluster encodes PYHIN proteins, which play important roles in sensing pathogen DNA [54], cytokine promoter induction in macrophages [55], and cell proliferation [56], all of which are prominent functions of KCs in the liver.

One of the most useful details provided by Hi-C is the identification of statistically significant contacts between enhancers and gene promoters, which underlie the promotion of expression by the enhancers [57,58]. Without such data, it is generally assumed that an enhancer regulates the closest expressed gene within a specific genomic size, usually 200 to 250 kb. Although our dataset provided this resource for all putative enhancers, we were particularly interested in identifying the contacts between SEs and gene promoters in our KC Hi-C map. SEs are thought to ensure a high level of expression of specific genes that play a significant role in functions related to cell phenotype [22]. Thus, we expected that identifying these contacts in our data would reveal the mechanisms regulating a core group of critical KC genes. We used published H3K27ac ChIP-seq data from KCs [59] to identify the SEs of KCs, and used FitHiC2 to identify the most significant contacts between KC SEs and the transcription start sites (TSS) of expressed genes in KCs (Table S8). We found that the median distance between these TSSs and their contacting SEs was 200 kb (Table S8). Hence, at least half of these contacts were likely missed without this direct evidence of contact between these loci. We found, as expected, that these genes were predominantly enriched for immune-related signaling pathways, including antigen processing and presentation and the NF-kappa B signaling pathway (Figure 4C). For instance, the *Zfp36l1* gene showed a significant interaction with an SE that is approximately 500 kb away (Figure 4D), well beyond the 200 kb to 250 kb conventionally expected range for enhancer/promoter contacts. Similarly, the *Klf3* gene interacted significantly with a SE located 400 kb away (Figure 4E). Both proteins play significant roles in KC function, with *Zfp36l1* involved in cellular metabolism [60] and *Klf3* playing a dominant role in NF-κB-mediated inflammation [61]. Notably, the interaction of the former with SE was not prominent in liver Hi-C data (Figure S12C and D).

Similarly, previous studies examining differential expression across several different tissue-resident macrophage populations identified a signature KC-specific gene set [62]. We found that nearly half of these KC-specific genes (144 out of 303) showed specific contacts with SEs in KCs (Table S9). Among these is *Clec4f*, one of the marker-defining genes of the KCs [63,64]. We find that this gene exhibited long-range contacts with 2 SEs that are 300 to 600 kb away (Table S8). We noted that earlier work identified a putative enhancer for the *Clec4f* gene in KCs located approximately 20 kb from its TSS [65]. Our data suggest that the regulation of this gene is more complicated and involves multiple distal SEs. The strict expression of this gene in KCs [65] (absent in other liver cells or extrahepatic macrophages) is likely owing to these chromatin interactions with distal SEs. In addition, we found that one of the most highly expressed groups of KC-specific genes, the members of complement C1q proteins (C1qa, C1qb, and C1qc), exhibited many distal contacts with an SE located around 450 kb away (Figure S13). A putative enhancer for these genes in a number of human immune cells were identified [66]; however, this enhancer location did not appear to be syntenic with the SE that we found contacted these genes in mouse KCs. This suggests that there is a KC-specific mechanism for regulating the *C1q* genes by specific chromatin structures that is important for systemic immune surveillance and pathogen elimination [67]. Overall, our maps enable the identification of a core group of KC genes, whose expression is likely owing to contacts with SEs identified in these Hi-C maps that can now be tested using additional functional assays.

We also noted that most KCs were located within zone 2 (between the periportal and pericentral regions) [68], and there is some suggestion that, similar to hepatocytes, KCs may also exhibit zonated expression. Thus, future work aimed at characterizing the spatial dependence of chromatin structure in the liver, together with transcriptomic data obtained from similar cells, could provide significant insights into the role of the local microenvironment of these cells and its influence on liver function.

Finally, we expected that our data may also be useful for illuminating differences in regulatory mechanisms among different types of macrophages [69,70]. While, to our knowledge, there are no Hi-C profiles of other tissue-resident macrophages directly isolated from native tissue, there is a profile of BMDMs induced from BM-derived monocytes *in vitro* [71]. This model system is often considered to exhibit many of the characteristics of macrophages in the blood [72,73]. Hence, we expected that a comparison between our KC Hi-C data and this published data may provide insights into liver-specific regulatory mechanisms for macrophages.

Interestingly, these maps exhibited a similar level of overall correlation (SCC of 0.82) to that between the KCs and our total liver sample (0.86), thus indicating a substantial level of differences in structure between these two macrophage types. In total, 12% of compartment bins and 42% of TAD borders (Figure S14A) were different between the two cell types. Genes in the A compartments in KCs and B compartments in BMDMs were enriched in pathways related to monoatomic cation (including iron) homeostasis and intracellular iron ion sequestration (Figure S14B), both of which are functions of KCs involved in recycling aged erythrocytes [74–76]. For example, *Bmp6* and *Gdf2* play a significant role in maintaining iron homeostasis and were present in the A compartments of KCs and the B compartments of BMDMs (Figure 4F). Further, the prominent scavenger receptors, *Colect12* (Figure 4F) and *Marco* (Table S10), were also present in the A compartments in KCs and the B compartments in BMDMs. Recent work has shown that scavenger receptor genes contribute to a higher accumulation of low-density lipoproteins in KCs, a unique function of the liver-resident KCs [63,77].

We identified significantly different regions of contact between the KCs and BMDMs using diffHic [21] and identified several instances that appear to underlie the differences in gene expression (Table S11). For instance, the *Slc40a1* gene, which encodes ferroportin and plays a pivotal role in iron metabolism in KCs [75,78], showed specific contact with a distal putative enhancer [53] only in the KC Hi-C map (Figure 4G). Additionally, the *Nr1h3* gene, which encodes the transcription factor LXRα [62], also exhibited distinct interactions with an enhancer (in fact, an SE) in the KC map (Figure 4G), a chromatin architectural feature that likely drives its spatiotemporal expression. LXRα has been suggested to be a lineage-determining transcription factor (LDTF) of KCs [79], and we indeed find that other LDTFs (*Spic*, *Id1*, *Id3*, and *Irf7* [65,80]) also showed significant interactions with distal SEs in KCs (Figure S15; Table S8). For example, *Id3* is essential not only for KC differentiation during embryonic development but also for their anti-tumor activity in adulthood [81]. Therefore, mapping such chromatin structural architectures (as achieved here) provides novel insights into the mechanistic basis underlying the expression and maintenance of the KC phenotype.

However, we also noted that this comparison enabled the identification of unique contacts only in the BMDM Hi-C data. For example, the *Itgam* gene, which encodes the BMDM-specific marker Cd11b [82], exhibited a much stronger contact with a putative enhancer [53] in the BMDM Hi-C data (Figure 4G). Thus, beyond informing regulatory mechanisms within KCs, our data provide insights into potential regulatory mechanisms in macrophages that function in other regions of the body, once similar data have been obtained from these regions.

## Discussion

The recent success of spatial transcriptomics methods in revealing previously unknown details of cellular functioning in tissues has driven significant interest in developing techniques that can provide complementary data at cellular resolution within native tissues [83]. Chromatin structure is well known to play a significant role in the regulation of gene expression. In particular, there are now several methods that can directly reveal regions of chromatin accessibility (notably ATAC-seq) from cells within tissues [84,85]. As increased chromatin accessibility is associated with active enhancers and promoters, these studies have provided molecular-level insights into the precise transcription factors that underlie the gene expression in tissues [84,86]. However, it is often not clear precisely which enhancer(s) regulate which gene(s) from this dataset. Therefore, the identification of chromatin contacts, using methods such as Hi-C, appears to be indispensable [87–89].

In this work, we provide the first Hi-C-based method to characterize chromatin structure within specifically defined cells in the tissue. We demonstrated that, in terms of the Hi-C procedure, our method yields data quality comparable to that obtained *in vitro* (Table 1) and has a similar level of efficiency to published single-cell Hi-C data (Table S2). As both LCM and Hi-C are widely used in many laboratories, we anticipate that our method will be rapidly adopted to provide direct information about the important chromatin contacts that underlie cell function in various contexts. For example, this method could be used to detect specific cells within the tumor microenvironment, such as immune cells in close proximity to malignant cells with unique phenotypes, to enable not only a more comprehensive understanding of these phenotypes but also their potential cell-cell interactions. Similarly, Histo-LCM-Hi-C could be used to resolve dynamic chromatin reorganization during organogenesis. For instance, isolating SOX2^+^ progenitor cells [90,91] at precise developmental stages within the subventricular zone of embryonic cerebral cortex could reveal how chromatin conformation contributes to the regulation of neurogenesis during mouse organogenesis.

We anticipate considerable interest in integrating Hi-C with other extant spatial-omics methods to provide a more comprehensive understanding of tissue functioning. In particular, our Hi-C method enables full characterization of the genome structure of phenotypically defined cell types. Spatial ATAC-seq approaches [90,92] provide highly complementary information: they typically identify open chromatin loci (∼ 1 kb in breadth) that can be used to identify transcription factor binding sites within highly localized regions of the tissue. Spatial transcriptomics can map gene expression of at least 10,000 genes within the spatial context of tissues [2,93], and spatial proteomics can be used to determine the abundance of ∼ tens of proteins per cell type within a tissue [94]. However, spatial ATAC-seq and most spatial transcriptomic approaches remain somewhat limited in spatial precision, identifying features within spots that may contain more than one cell. In addition, spatial proteomics is limited by the number of proteins that can be simultaneously detected in each cell. By contrast, a major limitation of LCM-based methods is throughput. Therefore, we believe that integrating the greater depth but lower breadth of LCM-based methods with the lower depth but greater breadth of existing spatial-omics methods will be more effective in resolving fundamental features of tissue function than any single method alone.

A particular noteworthy aspect of our method, even among spatial-omics methods, is its ability to characterize cells that constitute a significant minority of the total tissue cell population. This is a simple consequence of the basic LCM method for identifying and isolating specifically defined cells in the tissue. We believe that the ability to characterize rare cells within tissue will be an attractive feature that complements other high-throughput spatial-omics methods. We previously established a method to characterize the transcriptome of specifically defined cells with LCM from as few as 50 cells [95], and other researchers have characterized other molecular properties, including metabolites and proteins, using LCM from tens to hundreds of cells [96,97]. Thus, we envision that LCM-based methods will occupy a particularly valuable niche within the repertoire of spatial-omics methods by enabling a more detailed understanding of specific cell populations identified by coarser spatial approaches. Ultimately, such a level of understanding may be needed for all of the cells in the tissue to fully understand tissue behavior. However, until high-resolution, spatial, multi-modal methods are developed, the combination of high-resolution data from a few specific cells using LCM-based approaches, combined with lower-resolution, higher-throughput methods, may prove to be a powerful approach that can now be employed with present technology.

## Ethical statement

The experiments with mice were approved by the Institutional Animal Care and Use Committee (IACUC) at Shanghai Jiao Tong University (Approval No. A2020109).

## Supplementary Material

qzaf091_Supplementary_Data

## Data Availability

The raw sequence data have been deposited in the National Center for Biotechnology Information BioProject (BioProject: PRJNA1187272) at https://www.ncbi.nlm.nih.gov/bioproject. The data have also been deposited in the Genome Sequence Archive [98] at the National Genomics Data Center (NGDC), China National Center for Bioinformation (CNCB) (GSA: CRA024256), and are publicly accessible at https://ngdc.cncb.ac.cn/gsa/browse/CRA024256.

## CRediT author statement

**Yixin Liu:** Investigation, Methodology, Writing – original draft. **Min Chen:** Software, Formal analysis, Writing – review & editing. **Xin Liu:** Investigation, Methodology. **Zeqian Xu:** Formal analysis. **Xinhui Li:** Investigation. **Yan Guo:** Investigation. **Daniel M. Czajkowsky:** Conceptualization, Formal analysis, Writing – original draft. **Zhifeng Shao:** Conceptualization, Methodology, Writing – original draft. All authors have read and approved the final manuscript.
