# Supplementary material for: Histo-LCM-Hi-C Reveals 3D Chromatin Conformations of Spatially Localized Rare Cells in Tissues at High Resolution
Source: Genomics Proteomics Bioinformatics. 2025 Sep 30;24(1):qzaf091. doi: 10.1093/gpbjnl/qzaf091 (PMC13437044; doi:10.1093/gpbjnl/qzaf091)
Supplement: Supplementary file 1 [file qzaf091_supplementary_data.zip › Supplementary Material Captions.docx]

**Supplementary material**

**Figure S1 Evaluation of the efficiency of fixation protocol by MNase digestion results**

**A.** MNase digestion of mouse liver blocks after incubation with 1% paraformaldehyde for 10 min at RT results in a broad range of digested genomic DNA. **B.** A similar experiment but after incubation with 1% paraformaldehyde for 4 h at 17°C results in the digestion of genomic DNA into nucleosome-sized bands. **C.** MNase digestion of mouse liver blocks after incubation with fixation protocol in (B) and conventional fixation protocol in Hi-C.

**Figure S2 Evaluation of the efficiency of modified MboI reactions on crosslinked tissue samples**

**A.** and **B.** The DNA distribution obtained from liver tissue sections with thicknesses of 10 µm (A) and 20 µm (B), using the optimized crosslinking conditions and incubating with SDS and MboI under the typical conditions for standard Hi-C. **C.** Bar chart depicting the relative abundance of DNA in the indicated gel bands from the 10 μm sections without MboI treatment or the 10 μm, 20 μm or 50 μm thickness sections after MboI treatment. **D.** A similar experiment as (A) but with a longer SDS treatment time, which leads to a greater amount of well-digested DNA.

**Figure S3 Evaluation of the efficiency of modified T4 ligase reactions on crosslinked tissue samples**

**A.** Effectiveness of T4 ligation using the standard *in vitro* Hi-C conditions with optimized crosslinking and SDS treatment, showing a poor level of ligation. **B.** Optimized ligation conditions yielded a single sharp band in this agarose gel, reflecting effective ligation of the genomic DNA. **C.** A similar experiment as (A) on a 10 μm section of mouse brain, yielded a well-digested genomic DNA and a sharp band in the top of agarose gel.

**Figure S4 Evaluation of the quality of tissue section Hi-C libraries from crosslinked mouse liver and brain**

**A.** and **B.** The size distribution of the Hi-C libraries from tissue sections of liver (A) and brain (B) using the optimized conditions for tissue sections.

**Figure S5 Comparison of the Hi-C data from liver tissue sections with standard Hi-C data from AML 12 cells**

**A.** The proportion of valid pairwise reads in the liver tissue section Hi-C compared with published data from AML 12 cells. **B.** The ratio of intra-chromosomal pairwise reads to inter-chromosomal pairwise reads in liver tissue section Hi-C as well as that for the published standard Hi-C data from AML 12 cells.

**Figure S6 Correlation results between the tissue section Hi-C data of mouse liver and brain and published Hi-C data**

**A.** and **B.** Correlation results between tissue section Hi-C data and published tissue cell Hi-C data in 500 kb (A) and 40 kb (B) resolution.

**Figure S7 Examples of Hi-C libraries with abnormal or normal DNA fragment distribution**

**A.** and **B.** DNA fragment distribution obtained from Agilent 2100 Bioanalyzer analysis revealed no DNA (A) or abnormal DNA sizes (B) from less than 600 nuclear regions (270 intact cells). **C.** and **D.** DNA fragment distribution for a library that is appropriate for sequencing. Namely, such a distribution exhibits a single, smooth peak or a few closely distributed peaks within the range between 300 to 800 bp.

**Figure S8 Evaluation of reproducibility of KC Hi-C data and comparison of the Hi-C data from liver tissue sections with KC Hi-C data**

**A.** Correlation results among the tissue section Hi-C data of the KC replicates, bin size: 500 kb. **B.** The proportion of valid pairwise reads in the liver tissue section Hi-C, liver cell standard Hi-C data and KC Hi-C data (libraries from about ~ 300 KCs each).

**Figure S9 Comparison of the numbers of compartments, TADs, and chromatin loops obtained from different cell starting (KC) quantities**

**A.** Number of compartments obtained from different cell numbers of cells at 200 kb and 100 kb resolution. **B.** and **C.** TAD number (B) and loop number (C) identified from different numbers of cells at 80 kb and 40kb resolution.

**Figure S10 Ratio of high-confidence interactions determined from FitHiC2 in small-cell-number (KC) datasets and large-input Hi-C datasets**

**Figure S11 Comparison of the differences in the compartments between liver sections and the KCs**

**A.** Percentages of compartment bins that are of a different type. Those that are different are described according to their designation in the liver sections (A or B) that are different from those in the KCs (B or A). **B.** Box plots showing the observed / expected contact strength in inter-compartment between the same compartment type in liver tissue section Hi-C data and the KC Hi-C data (***, *P* < 0.001, Wilcoxon rank sum test). **C.** GO analysis results of the genes located the A compartment in the liver and B in the KCs, most of which are associated with metabolic pathways that function in hepatocytes.

**Figure S12 Specific contacts between predicted enhancer and functional genes in the KC Hi-C map**

**A.** and **B.** Visualization of contact maps generated from liver tissue section Hi-C data (A) and KC Hi-C data (B) in Chr1: 172.8 Mb–175Mb, indicating stronger contacts between a putative enhancer and the *Ifi200* family gene cluster in KC. ENCODE cCREs, Refseq genes and ReMap ChIP-seq are shown below. **C.** and **D.** Visualization of the contact maps generated from liver tissue section Hi-C data (C) and KC Hi-C data (D) in Chr12: 79.4 Mb-80.8 Mb, indicating stronger contacts between a putative SE of KCs and the *Zfp36l1* gene in KC Hi-C data. The Refseq genes are shown below. Resolution: 40 kb.

**Figure S13 Contact between the KC-specific genes and their distant SE**
Visualization of contact maps generated from KC Hi-C data in Chr4 showing the contacts between the *C1qb*,*C1qc* and *C1qa* genes and a distant SE. Shown below are the H3K27ac ChIP-seq data of the KCs, Refseq genes and the SE (black bar). Resolution: 20 kb

**Figure S14 Comparison of the differences in the TAD and compartments between KCs with BMDM**

**A.** Venn diagram of the TAD borders of the KCs and BMDMs. **B.** GO analysis results of the genes located in the A compartment in the KCs and B compartment in the BMDMs.

**Figure S15 Contact between the KC lineage-determining TF genes and their distant SEs**
**A.**–**E.** Visualization of contact maps generated from the KC Hi-C data showing the loci of the genes encoding the lineage-determining transcription factors *Nr1h3* (A), *Spic* (B), *Id1* (C), *Id3* (D), and *Irf7* (E). The white dotted boxed regions highlight the contact between the genes and SEs. Shown below are the H3K27ac ChIP-seq data of the KCs, Refseq genes and the SE (black bar). Resolution: 20 kb.

**Table S1 Filter results of the sequencing data from Histo-LCM-Hi-C library (mouse liver)**

**Table S2 Filter results of the sequencing data from KC Hi-C libraries and the contact per cell in published single cell Hi-C**

**Table S3 Filter results of the sequencing data from published low input Hi-C data**

**Table S4 Significant contacts from FitHiC2 in the small-cell-number (KC) datasets before and after down-sampling**

**Table S5 TADs annotation results of KC**

**Table S6 Genes located in the A compartment in the KCs and B in the liver**

**Table S7 DiffHic results between KC and total liver**

**Table S8 Predicted contacts of SE-gene in KC from FitHi-C2**

**Table S9 KC-specific genes list**

**Table S10 Genes located in the A compartment in the KCs and B in the BMDMs**

**Table S11 DiffHic results of contacts between putative enhancer and *Slc40a1*, *Nr1h3*, and *Itgam* genes between KC and BMDM**
