# Supplementary material for: Histo-LCM-Hi-C Reveals 3D Chromatin Conformations of Spatially Localized Rare Cells in Tissues at High Resolution
Source: Genomics Proteomics Bioinformatics. 2025 Sep 30;24(1):qzaf091. doi: 10.1093/gpbjnl/qzaf091 (PMC13437044; doi:10.1093/gpbjnl/qzaf091)
Supplement: Supplementary file 1 [file qzaf091_supplementary_data.zip › Table S3.docx]

**Table S3 Filter results of the sequencing data from published low input Hi-C data**

|  | **2018, Diaz et al.** | **2020, Zhang et al.** | **2020, Zhang et al.** |
| --- | --- | --- | --- |
| Total reads | 86,616,647 | 79,596,960 | 93,469,253 |
| MAPQ ≥ 30 count | 57,057,214 | 45,191,240 | 71,513,055 |
| Duplicate removed | 29,559,433 | 34,405,720 | 21,956,198 |
| Nonspecific | 2,749,046 | 9,069,175 | 6,630,422 |
| Valid pairs | 26,810,387 | 25,336,545 | 15,325,776 |
| Cis-interaction | 22,811,714 | 19,896,310 | 11,764,130 |
| Cell number | 1000 | 500 | 200 |
| Valid / cell | 26,810 | 50,673 | 76,629 |
