# Supplementary material for: Histo-LCM-Hi-C Reveals 3D Chromatin Conformations of Spatially Localized Rare Cells in Tissues at High Resolution
Source: Genomics Proteomics Bioinformatics. 2025 Sep 30;24(1):qzaf091. doi: 10.1093/gpbjnl/qzaf091 (PMC13437044; doi:10.1093/gpbjnl/qzaf091)
Supplement: Supplementary file 1 [file qzaf091_supplementary_data.zip › Table S4.docx]

**Table S4 Significant contacts from FitHiC2 in the small-cell-number (KC) datasets before and after down-sampling**

|  | **Before down-sampling** | **After down-sampling** | **Overlap** | **Before down-sampling/**  **overlap%** | **After down-sampling/**  **overlap%** | |
| --- | --- | --- | --- | --- | --- | --- |
| Contacts  (q-value < 0.05) | 4,690,457 | 4,408,583 | 4,310,396 | 91.90% | | 97.77% |
| Contacts  (q-value < 0.01) | 3,011,875 | 2,836,947 | 2,760,925 | 91.67% | | 97.32% |
| Contacts  (q-value < 0.005) | 2,681,144 | 2,512,675 | 2,456,975 | 91.64% | | 97.78% |
